# Comparison of Isotonic Activation of Cell Volume Regulation in Rat Peritoneal Mesothelial Cells and in Kidney Outer Medullary Collecting Duct Principal Cells

**DOI:** 10.3390/biom11101452

**Published:** 2021-10-03

**Authors:** Galina S. Baturina, Liubov E. Katkova, Claus Peter Schmitt, Evgeniy I. Solenov, Sotirios G. Zarogiannis

**Affiliations:** 1 Institute of Cytology and Genetics, SB RAS, 630090 Novosibirsk, Russia; baturina@bionet.nsc.ru (G.S.B.); ile@bionet.nsc.ru (L.E.K.); eugsol@bionet.nsc.ru (E.I.S.); 2 Center for Pediatric and Adolescent Medicine, University Hospital Heidelberg, 69120 Heidelberg, Germany; clauspeter.schmitt@med.uni-heidelberg.de; 3 Department of Physiology, Novosibirsk State University, 630090 Novosibirsk, Russia; 4 Department of Data Acquisition and Analysis, Novosibirsk State Technical University, 630087 Novosibirsk, Russia; 5 Department of Physiology, Faculty of Medicine, School of Health Sciences, University of Thessaly, 41500 Larissa, Greece

**Keywords:** cell volume regulation, kidney principal cells, mesothelial cells, organic osmolytes, osmotic stress

## Abstract

In disease states, mesothelial cells are exposed to variable osmotic conditions, with high osmotic stress exerted by peritoneal dialysis (PD) fluids. They contain unphysiologically high concentrations of glucose and result in major peritoneal membrane transformation and PD function loss. The effects of isotonic entry of urea and myo-inositol in hypertonic (380 mOsm/kg) medium on the cell volume of primary cultures of rat peritoneal mesothelial cells and rat kidney outer medullary collecting duct (OMCD) principal cells were studied. In hypertonic medium, rat peritoneal mesothelial cells activated a different mechanism of cell volume regulation in the presence of isotonic urea (100 mM) in comparison to rat kidney OMCD principal cells. In kidney OMCD cells inflow of urea into the shrunken cell results in restoration of cell volume. In the shrunken peritoneal mesothelial cells, isotonic urea inflow caused a small volume increase and activated regulatory volume decrease (RVD). Isotonic myo-inositol activated RVD in hypertonic medium in both cell types. Isotonic application of both osmolytes caused a sharp increase of intracellular calcium both in peritoneal mesothelial cells and in kidney OMCD principal cells. In conclusion, peritoneal mesothelial cells exhibit RVD mechanisms when challenged with myo-inositol and urea under hyperosmolar isotonic switch from mannitol through involvement of calcium-dependent control. Myo-inositol effects were identical with the ones in OMCD principal cells whereas urea effects in OMCD principal cells led to no RVD induction.

## 1. Introduction

The maintenance of a constant cell volume in the face of extracellular and intracellular osmotic perturbations is an issue faced mainly by epithelial cells. Most cells respond to osmotic challenges by activating specific membrane transport processes that serve to return cell volume to its normal, initial value [1,2]. These processes are essential for normal cell function and survival. The adaptive mechanism of recovery after cell shrinkage in a hypertonic environment is termed regulatory volume increase (RVI), while recovery after cell swelling in a hypotonic environment is termed regulatory volume decrease (RVD) [3,4,5]. The key process of RVI and RVD is the gain or loss of intracellular osmotically active molecules (osmolytes) followed by an influx or efflux of extracellular water, respectively. Thus, the molecular counterparts of cell volume regulation are electrolyte transporters, transport, and synthesis of organic osmolytes allowing the passage of water according to the osmotic gradient [5,6].

The renal collecting tubule is the key regulator of the extracellular volume and solute composition of the organism. Since the final composition of the urine is determined in the collecting duct, where they are exposed to a wide range of osmotic gradients (from 100–1400 mOsm/Kgr H_2_O), principal cells are the major cell population of the collecting ducts and are critical in mediating the fine tuning of urine solute and water concentrations [7,8,9,10]. Therefore, these cells have the most effective mechanisms to maintain their viability and functionality in an extracellular environment with a highly varying osmotic pressure [10,11,12]. They are the most fit cell type for comparison when other epithelial cells are evaluated regarding their cell volume regulation potential.

The characteristics of the cell volume regulation mechanisms in mesothelial cells vesting the pleural and peritoneal cavities are largely unexplored. The only study that has investigated the osmotic water permeability of human pleural mesothelial cells when compared to human malignant pleural mesothelial cells has shown that benign mesothelial cells have a significantly higher osmotic water permeability than malignant counterparts in baseline as well as after hyperglycemia challenge [13]. In both cells, the osmotic water permeability was significantly reduced after aquaporin-1 inhibition by HgCl_2_. The osmotic water permeability of the benign mesothelial cells was 0.005 cm/s which is much lower compared to OMCD principal cells, which have an osmotic water permeability of 0.12 cm/s [10]. In patients with pleural effusions of different etiologies, effusion osmolality ranges from 240 to 340 mOsm/kgr H_2_O. Pleural mesothelial cells in pathological conditions (pleural infection or malignancy) have to regulate their volume and adjust to aniso-osmolality [14]. Likewise, peritoneal mesothelial cells are also exposed to variable osmotic conditions with ascites formation. A massive increase in osmolality occurs in patients undergoing peritoneal dialysis (PD). Next to hemodialysis, PD is the standard renal replacement therapy in patients with acute kidney injury and in patients with chronic end stage renal disease. In these patients, high volumes of PD fluids are repeatedly infused into the peritoneal cavity [15]. To achieve adequate removal of water (ultrafiltration) from the circulation of the mostly oligo- anuric patients, PD fluids contain high concentrations of glucose creating a hyperosmolar environment ranging between 344 and 511 mOsm/kgH_2_O, depending on the glucose concentration [16]. The hyperosmolarity only partially dissipates until the next PD fluid exchange. In addition, conventional PD solutions have an acidic pH and contain high amounts of toxic glucose degradation products (GDP), while so called biocompatible PD fluids, which separate the glucose from the buffer during sterilization and storage, have a neutral to physiological pH and are largely devoid of GDP. Despite these improvements, both types of PD fluids still induce severe transformation of the peritoneal membrane during chronic PD, most importantly peritoneal fibrosis and hypervascularization that ultimately lead to ultrafiltration failure [17,18,19]. With both types of PD fluids, the peritoneal mesothelial cell monolayer is progressively lost. The mesothelial cells detach and undergo apoptosis and mesothelial-to-mesenchymal transition (MMT). MMT cells invade the submesothelial space where they further aggravate the tissue transformation process [20].

Taken together, alterations of osmolality are a key driver of mesothelial cell damage. Understanding exactly how mesothelial cells change their volume while responding to the severe hyperosmotic stress should provide important insights on osmoprotective mechanisms and inform the development of more biocompatible solutions. To this end, the current work studied the plasma membrane transport and cell volume changes on isotonic gradients of urea and myo-inositol in rat peritoneal mesothelial compared to kidney OMCD principal cells in a hypertonic environment.

## 2. Materials and Methods

### 2.1. Animals

Adult Wistar rats (200–250 gr) were bred and maintained in standard housing conditions at the Animal Facility of the Institute of Cytology and Genetics (Novosibirsk, Russia). Rats were fed a standard pellet chow and had free access to water. During the experiments, the animals were anaesthetized with intraperitoneal (i.p.) injections of sodium thiopental (10 mg/kg body wt) and were decapitated and, immediately thereafter, their kidneys were surgically removed.

### 2.2. Cell Culture

Primary rat peritoneal mesothelial cells and primary principal cells from micro-dissected fragments of outer medulla collecting ducts (OMCD) were used in the study. Cell culture was performed in RPMI-1640 cell medium supplemented with 10% BCS, 1% antibiotics, and 1% L-glutamine in a 5% CO_2_ humidified incubator at 37 °C. All chemicals were purchased from Sigma-Aldrich (St. Louis, MO, USA) unless otherwise stated.

### 2.3. Renal Outer Medullary Collecting Duct Suspension Preparation

Renal OMCD suspension was prepared as described previously [8]. Briefly, the kidney outer medulla zone was placed in a syringe containing ice-cold, calcium-free PBS solution (PBS: 137 mM NaCl, 4.7 mM Na_2_HPO_4_, 2.7 mM KCl, 1.5 mM KH_2_PO_4_, 0.5 mM MgCl_2_, 5.5 mM glucose, 0.1 mM CaCl_2_, pH = 7.4). Subsequently, the tissue was squeezed through a needle (0.45 mm i.d.). The resulting suspension was filtered through a nylon mesh, diluted 10 times with MEM culture medium and centrifuged (100 g, 10 min, 4 °C), then 0.1 mL of suspension transfer to cover glass covered with Poly-L-lysine (Sigma Aldrich, St. Louis, MO, USA). The fragments were maintained in MEM cell medium that has normal osmolality.

### 2.4. Isolation of Peritoneal Mesothelial Cells

Pieces of parietal peritoneum (200–300 mg) were taken in sterile conditions and incubated with a minimal volume of 0.25% trypsin (Trypsin-EDTA solution, Sigma, USA) for 5 min at 37 °C. Then the tissue was transferred to 10–15 mL of calcium free PBS and shaken for 2 min. The residual tissue was removed from the solution and the cell suspension was centrifuged at 1500 g for 5 min. The procedure was repeated 3 times. The cellular sediment was resuspended in 15 mL of culture medium RPMI-1640 (Sigma Aldrich, USA) with 20% FCS (Sigma Aldrich, USA). A 2 mL aliquot of cell suspension was transferred to a 35 mm petri dish with cover glasses (22 × 22 mm) (BRAND^®^ cover glass, Merck KGaA, Darmstadt, Germany) and was grown to 70–90% of confluence in CO_2_ incubator in RMPI cell medium (37 °C, 5% CO_2_).

### 2.5. Experimental Conditions Rationale

**Figure 1:**
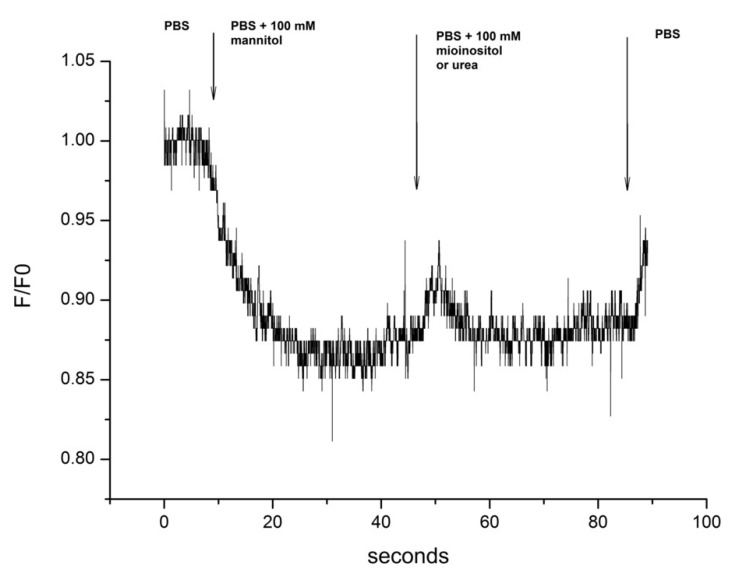
Schematic of the experimental design depicting the changes of the extracellular medium from PBS to PBS + 100 mM mannitol and subsequently to PBS + 100 mM of myo-inositol or urea. F/F0: Ratio of Fluorescence intensity to the initial fluorescence at time 0 s.

Incubation of cells in hypertonic medium causes their shrinkage, subsequently inducing RVI. Typically, RVI is mediated by the increase of the intracellular concentration in osmotically active agents such as electrolytes and non-ionic molecules. Urea and myo-inositol are the most important intracellular electroneutral organic osmolytes. The aim of these experiments was to measure the effects of urea and myo-inositol inflow on the cell volume in a hypertonic environment. The schematic of the experiment design is shown in Figure 1.

### 2.6. Water Permeability Measurements

The water permeability was calculated from the value of the initial rate of cell swelling after changing the osmotic pressure of the medium from 380 to 280 mOsm/kg. The hypertonic medium (380 mOsm/kg) where the cells were balanced before the measurement of water permeability was created by the addition of 100 mM mannitol in PBS (that has normally 280 mOsm/kg). Fluorescence measurements of cell volume were performed by the calcein quenching method as previously described [10,13,21]. Cell volume changes were expressed as relative values of calcein fluorescence, an established surrogate of the cell volume fluctuations [22]. The cells were loaded with the fluorescent probe Calcein-AM (5 μM, 20 min at 37 °C; Invitrogen, CA, USA). The fluorescence of calcein was continuously measured with a LED light source, through a Zeiss filter set #09 (BP 450–490 nm excitation, FT 510 nm dichroic mirror, LP 515 nm emission), a photomultiplier detector with a pinhole diaphragm in order to be able to select the cells of interest and with a digital oscilloscope ACK-3102 (Actacom^®^, RF Moscow, Russian), and saved on a PC. The data acquisition rate was set to 10 ms. A superfusion chamber was constructed as an acrylic block with a T-shape current of cell medium. This design makes a fast change of superfusion medium feasible and minimizes the perturbations of the specimens. The flow rate of the perfusate was set to 10 mL/min, which resulted in a complete solution exchange in the area of interest in less than 100 msec. The chamber was mounted on the stage of an inverted microscope (Axiovert 40, Zeiss, Germany; objective lens with 40× magnification; numerical aperture 0.65; thermal stabilization at 36.8 ± 0.2 °C). The water permeability was calculated from the rate of the cell volume changes under the osmotic challenge on the basis of the equation [23]: dV/dt = −AV_w_P_f_▽Φ. The osmotic water movement is the net flow of volume across a cell membrane in response to osmotic pressure dV/dt = −P_f_AV_w_∆C. The permeability coefficient can be calculated from the slope (K_r_) of the linear plot [10]:

P_f_ = K_r_ [AV_w_(C_in_ − C_out_)]^−1^. (1)

where (dV/dt) is the rate of cell volume change, P_f_ is the osmotic water permeability coefficient, A is the surface area which is significant for water exchange, ΔΦ is the osmotic pressure difference, ΔC is the osmotic concentration difference, and V_w_ is the partial molar volume of water.

In the water permeability measurements, the cells were incubated in a solution of PBS supplemented with 100 mM of mannitol until the fluorescent signal became stable and then the solution was rapidly replaced with normal PBS (280 mOsm kg^−1^) and the apparent water permeability was measured.

### 2.7. Measurement of the Effect of Urea and Myo-Inositol on Cell Volume

The cells were balanced in hypertonic medium that was created by the addition of 100 mM of mannitol in PBS (380 mOsm/kg) before the measurement of the effect of isotonic 100 mM urea or myo-inositol in PBS solution (380 mOsm/kg). The parameters of the cell volume changes as a result of isotonic urea or myo-inositol entry that were measured were the initial rate of cell volume change and the characteristic time of RVD.

### 2.8. Measurement of the Intracellular Calcium

Cells were loaded with the Fluo 4 AM indicator (10 mM) for 30 min at 37 °C and were then placed on a microscope stage with thermal stabilization at 36.8 ± 0.2 °C in PBS solution. Emitted fluorescence intensities were obtained using the experimental setup described above (PMT with a pinhole diaphragm through a Zeiss filter set #09).

### 2.9. Statistical Analyses

Data are presented as means ± SE. Statistical significance was evaluated using unpaired *t* test when comparing two variables. A value of *p* < 0.05 was considered significant.

## 3. Results

### 3.1. Experiments of Rat OMCD Principal Cells on Isotonic Myo-Inositol and Urea Switch in Hypertonic Environment

**Figure 2:**
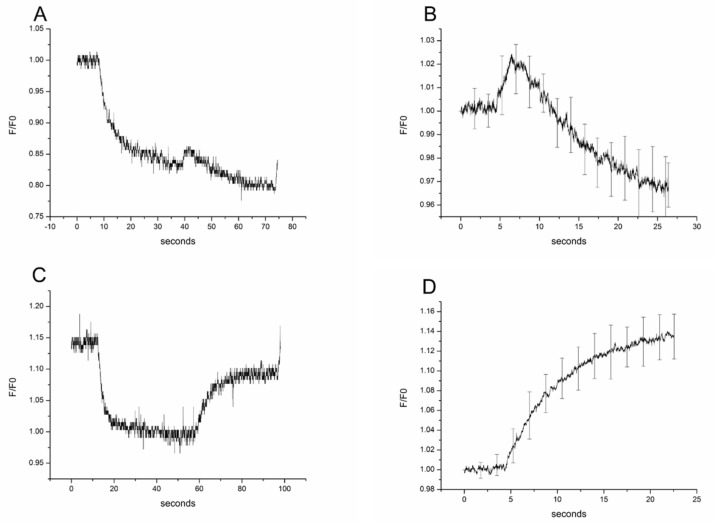
Effect of isotonic gradients (100 mM) of myo-inositol and urea on the cell volume of rat primary OMCD principal cells. A typical recording profile with myo-inositol incubation is given in (**A**) and the profile of the mean and SE of 18 experiments in (**B**). Typical recording profile of rat primary outer medullary collecting duct principal cells incubated with urea (**C**) and profile of mean and SD of 18 experiments (**D**). The (**B**,**D**) are the second part of the curves of (**A**,**C**) respectively, reflecting cell volume changes. They are normalized to the starting volume and the mean values of every point are used to get the mean profiles.

Primary rat kidney OMCD principal cells in experiments in a hypertonic environment with fast isotonic switch from mannitol to myo-inositol resulted in cell swelling within 2 s and a subsequent RVD activation (typical plot shown in Figure 2A). The characteristic time of RVD and thus cell volume decrease was 10.6 ± 0.13 s (Figure 2B; *n* = 18). This effect was not seen, however, during the isotonic switch from mannitol to urea (typical plot shown in Figure 2C). In this case, urea influx caused cell swelling that significantly restored the initial cell volume of the cells to nearly 90% and was not accompanied by RVD (Figure 2D; *n* = 18).

### 3.2. Experiments of Rat Parietal Peritoneum Mesothelial Cells on Isotonic Myo-Inositol and Urea Switch in Hypertonic Environment

**Figure 3:**
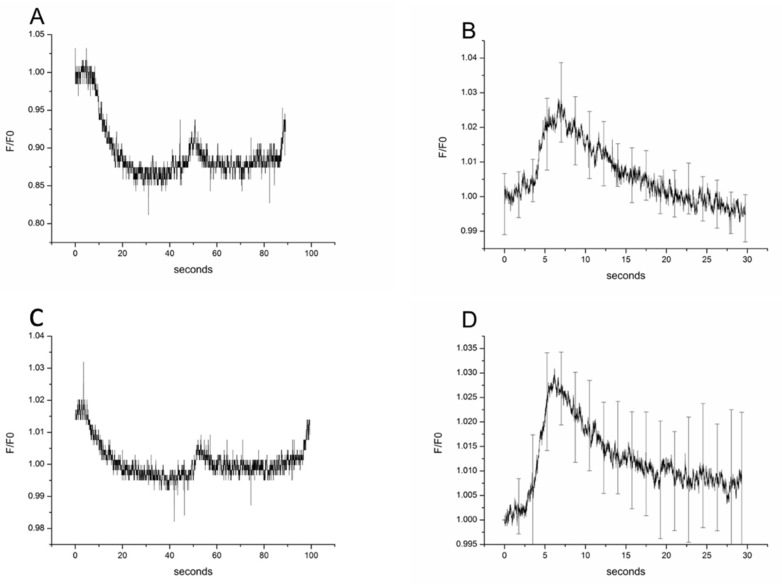
Effect of isotonic gradients (100 mM) of myo-inositol and urea on the cell volume of rat primary peritoneal mesothelial cells. A typical recording profile with myo-inositol incubation is given in (**A**) and the profile of the mean and SE of 18 experiments in (**B**). Typical recording profile of rat peritoneal mesothelial cells incubated with urea (**C**) and profile of mean of 18 experiments (**D**). The (**B**,**D**) are the second part of the curves of (**A**,**C**) respectively, reflecting cell volume changes. They are normalized to the starting volume and the mean values of every point are used to get the mean profiles.

Primary rat parietal peritoneal mesothelial cells swelled after the isotonic change of mannitol to myo-inositol within 2 s and then the mechanism of RVD led to cell volume decrease, as shown in Figure 3A. The kinetics of RVD mechanism was 10.5 ± 0.2 s (*n* = 18; Figure 3B), which was similar to the results obtained in the OMCD principal cells. A similar cell reaction was observed when the isotonic switch involved a change from mannitol to urea, where urea entered the cells and led to cell swelling within 2 s, which was followed by RVD (characteristic plot in Figure 3C). The characteristic time of RVD and thus cell volume decrease in this case was 6.2 ± 0.10 s (Figure 3D; *n* = 18). Taken together, the RVD was signigicantly faster in the case of urea compared to myo-inositol in rat primary parietal peritoneal mesothelial cells.

### 3.3. Comparison of RVD Kinetics of Myo-Inositol between Rat Primary OMCD Principal and Parietal Peritoneal Mesothelial Cells

The original rate of cell swelling in isotonic myo-inositol and urea was faster in primary rat kidney OMCD principal cells in comparison to parietal peritoneal mesothelial cells as it can be seen by the coefficients of the relative cell volume linear regression. In fact, evaluation of the initial rate of cell swelling showed that the principal cells have higher permeability for both myo-inositol and urea and that urea enters both cell types faster than myo-inositol. More specifically, for myo-inositol in principal cells the regression coefficient was r = 0.0088 ± 0.00018 (±0.000156 95%CI) and in peritoneal mesothelial cells r = 0.0069 ± 0.00097 (±0.0000841 95%CI). Regarding urea in principal cells r = 0.0194 ± 0.00033 (±0.000286 95%CI) and in peritoneal mesothelial cells r = 0.0109 ± 0.00018 (±0.000156 95%CI).

### 3.4. Comparison of RVD Kinetics Relative to Intracellular Calcium between Rat Primary OMCD Principal and Parietal Peritoneal Mesothelial Cells

**Figure 4:**
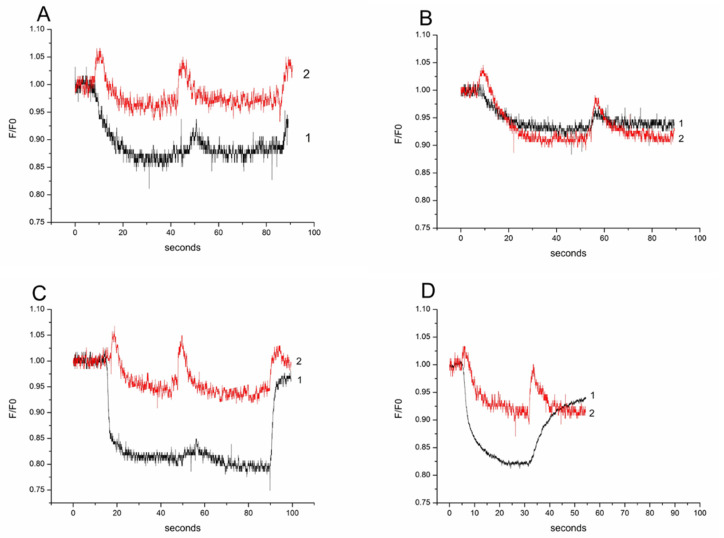
Effect of isotonic gradients (100 mM) of myo-inositol and urea on the cell intracellular calcium. Synchronized profiles for Calcein (black plot-1 demonstrating cell volume) and Fluo4 (red plot-2 demonstrating [Ca^2+^]_i_) in rat primary mesothelial cell experiments with (**A**) myo-inositol and (**B**) urea. Synchronized profiles for Calcein and Fluo4 in rat primary outer medullary collecting duct principal cells in experiments with (**C**) myo-inositol and (**D**) urea.

Changes in the concentration of intracellular calcium ([Ca^2+^]_i_) during regulatory volume changes were assessed using the fluorescent probe Fluo4. Every tonicity change in the extracellular medium in our experiments disturbed the cell volume. In these instances, spikes of [Ca^2+^]_i_ were observed as the cells were challenged by hypertonic medium that contained mannitol, or when the mannitol content of the medium was changed for either myo-inositol or urea with the same osmotic concentration. In the experiments with synchronized fluorescence profiles of calcein and Fluo-4, the concurrent changes in cell volume (calcein) and [Ca^2+^]_i_ (Fluo-4) could be seen, demonstrating that the peaks of calcium correspond to the cell volume changes (Figure 4). Mesothelial cells exhibit RVD for myo-inositol, similar to primary OMCD principal cells, and exhibit RVD for urea challenge, which is different than the reaction in principal OMCD cells, where swelling without RVD occurs in primary OMCD principal cells. Thus, we speculate that there could be differences in the urea transporters.

## 4. Discussion

Cells that face osmotic challenges due to changes in extracellular osmolality adapt their volume through modifying the intracellular solute content so as to adapt by either inducing RVD (in case of cell swelling) or RVI (in case of cell shrinkage) [2]. These mechanisms are mediated by membrane transport activation as well as metabolic processes that result in net solute loss or gain and return to the initial resting cell volume [2,5]. During these transport processes, the intracellular solute content and volume is changed [23]. Urea and myoinositol are two key osmolytes in kidney physiology and pathophysiology [5,24,25]. Urea is a critical molecule for the nephron countercurrent exchange mechanism that defines the capacity of the nephrons to concentrate urine and thus avoid excess water loss. In this aspect, it serves as one of the osmolytes that aids in the maintenance of the hyperosmotic environment in the renal medulla interstitium [26,27]. The cells of the renal medulla also contain high concentrations of osmolytes like myo-inositol, i.e., organic osmolytes produced by the cells in order to tolerate great osmotic gradients without affecting intracellular protein functions as it is the case with high ionic strength [3,28]. Therefore, the cells of the renal medulla are the best model to study the effects of osmolytes in cell volume regulation since tolerance to steep changes in the extracellular osmolality is an integral part of their homeostasis. All other cells of the human body may experience hyperosmolality under certain conditions, but obviously their volume regulation mechanisms should be less powerful. During persistent hyperosmolar conditions, the inorganic ions need to be replaced by compatible electrically neutral organic osmolytes that do not disturb protein function [1]. Urea and inositol could be identified as important electroneutral organic osmolytes, of which transmembranous transport could be involved in volume regulation [2,28].

PD treatment is based on the infusion of glucose rich, hyperosmolar PD fluids in the peritoneal cavity in order to remove excess water together with ions and metabolic byproducts (such as urea). In this setting the peritoneal mesothelial cells are chronically exposed to fluctuating but persistently hyperosmolar conditions, a key factor of mesothelial cell degradation. Nonetheless their cell volume regulation properties have scarcely been studied. Under hyperosmolar stress MeT-5A mesothelial cells have lower water permeability than principal OMCD cells, mediated mainly by AQP1 function [13].

We now demonstrate that parietal peritoneal mesothelial cells have urea and myo-inositol permeability, but at lower rates than principal OMCD cells. In both cell types the entry of urea is faster than that of myo-inositol and in both cell types myo-inositol gradients caused cell swelling and induced RVD. In contrast, urea induced similar reactions in peritoneal mesothelial cells, while the RVD response was absent in the principal OMCD cells, since they underwent a swelling process that allowed them to recover to their initial volume. The transport mechanism of urea in the principal OMCD cells is not well understood and it is not clear why RVD activation was not achieved, but it may be due to the fact that these cells are physiologically exposed to high urea concentrations and may have developed adaptative protection mechanisms that are not present in cells such as mesothelial cells.

Another finding of interest is the comparable intracellular calcium response to the isotonic switch of osmolytes in both cell types. The short intracellular calcium spikes were similar. They reflect activation of calcium-dependent processes including some control cell permeability and cell volume regulation [29]. This finding deserves more in-depth investigation in order to assess the calcium reaction related differences in the molecular mechanisms involved in the transport of osmolytes in two cell types.

Myo-inositol intracellular concentrations increased progressively in mesothelial cells in response to hypertonic stress, similar to PD conditions. At the molecular level this occurs by increasing the levels and activity of the sodium/myo-inositol cotransporter [30]. The higher urea than myo-inositol permeability rate in mesothelial cells could be explained by the fact that urea enters the cells by means of facilitated diffusion, whereas myo-inositol enters by means of secondary active transport, which is limited by the abundance of substrate and membrane cotransporter lifetime. The distribution of urea transporters in mesothelial cells is currently unknown but is of interest, since urea is osmotically active and a surrogate parameter of small solute toxin removal by PD, i.e., a biomarker of PD efficiency. We provide essential information of key response mechanisms of peritoneal mesothelial cells to hyperosmolality compared to OMCD cells, as present for example in patients with ascites. To better understand the mesothelial cell type specific response in the setting of PD, experiments including high extracellular glucose exposure should be amended. On the other hand, molecular weight of glucose and myo-inositol is identical, and both are cotransported with sodium, suggesting similar kinetics with glucose as shown here for myo-inositol.

## 5. Conclusions

We provide the first evidence that peritoneal mesothelial cells under isotonic switching in hyperosmolar conditions exhibit significant RVD reactions in response both in the case of myo-inositol and urea. Myo-inositol effects were identical with the ones in OMCD principal cells, whereas urea effects in OMCD principal cells led to no RVD induction. Future studies should aim at identifying the molecular counterparts of these fast processes and the translational potential of these findings in disease settings and treatments such as PD.

## Data Availability

Data can be made available by the corresponding author at reasonable request.
